# Beyond the dose-volume histogram: a critical appraisal of normal tissue complication probability modelling for osteoradionecrosis of the jaw and a strategic framework for clinical translation

**DOI:** 10.3389/fonc.2026.1893594

**Published:** 2026-07-10

**Authors:** Jaymit Patel

**Affiliations:** Leeds Dental Institute, Leeds Teaching Hospitals Trust, Leeds, United Kingdom

**Keywords:** head and neck cancer, machine learning, normal tissue complication probability, oral health, osteoradionecrosis, radiotherapy, survivorship

## Introduction

Head and neck cancer (HNC) has a substantial global burden, with an estimated 880,000 new cases worldwide each year (1–3). Radiotherapy (RT) remains the cornerstone of treatment, used in approximately 75% of cases, either as primary or adjuvant therapy in combination with surgery and systemic agents (3). The therapeutic success of RT in HNC, however, is inseparable from its toxicity profile, which includes xerostomia, dysphagia, trismus, and osteoradionecrosis (ORN).

ORN is characterised by radiation-induced hypoxia, hypovascularity, and hypocellularity of bone and soft tissue, leading to loss of cortical bone integrity, chronic non-healing, and progressive oral dysfunction (4, 5). Its incidence of 7–10% belies the severity of its consequences: advanced-grade disease may complex treatments and lengthy care pathways to achieve cure. Even lower-grade disease carries substantial quality of life burden (6, 7). The rising incidence of HPV-associated oropharyngeal cancers, which disproportionately affect younger, otherwise healthy individuals who enjoy prolonged survival following RT, has intensified the imperative to prevent ORN (8–10). This population faces a lifetime cumulative risk that exceeds that estimated from cross-sectional analyses conducted at fixed post-treatment intervals.

Normal tissue complication probability (NTCP) modelling offers a quantitative framework to translate pre-treatment dosimetric and clinical parameters into individualised risk estimates, with potential applications spanning treatment plan optimisation, patient stratification for proton therapy, and surveillance intensity stratification (11, 12). The field has advanced considerably over the past five years, from early cross-sectional case-control analyses (13) through the first validated NTCP model for ORN (14), to multi-institutional consortium efforts (15) and novel time-to-event approaches (16). Deep learning methods applied to three-dimensional dose distribution maps have further expanded the methodological repertoire (17).

Yet for all this methodological innovation, the clinical implementation of ORN NTCP models remains limited. This perspective argues that three structural deficiencies: insufficient cohort diversity, inadequate characterisation of RT protocol heterogeneity, and the consistent under-representation of oral health variables. We provide a narrative synthesis of the published literature, and in doing so propose that the above limitations constrain both the validity and the generalisability of existing models. We propose a strategic framework to address each, grounded in the current evidence base and oriented towards meaningful clinical translation. The scope of this is to provide a critical perspective on the literature in a manner that represents narrative opinion, rather than an analysis of the methodological robustness of studies. Any study reporting on NTCP of osteoradionecrosis in head and neck cancer was considered was part of the narrative synthesis. Articles not published in the English language (without an English language translation) were not included. There were no publication date limits imposed on the range of literature considered. Research articles not explicitly and solely reporting on NTCP modelling of osteoradionecrosis in head and neck cancer were not considered. The inclusion criteria are broad to align with the narrative methods.

## The current evidence base: achievements and limitations

### Established predictors and model architecture

The landmark NTCP model developed by van Dijk et al. (14) on a cohort of 1,259 patients treated at MD Anderson Cancer Center (MDACC) between 2005 and 2015 identified D30% (the minimum dose received by the most irradiated 30% of the mandibular volume) and pre-RT dental extraction as the two most discriminative independent predictors of any-grade ORN, yielding an area under the receiver operating characteristic curve (AUC) of 0.78 on the training cohort and 0.75 on the held-out validation set (14). This work established that mandibular dose-volume metrics across a broad ranged (2% to D98%, V15Gy to V70Gy) were all significantly associated with ORN in univariable analysis, but that the model combining D30% with dental extraction status provided robust and reproducible predictive information. The subsequent PREDMORN multi-institutional study, incorporating 3,928 patients from eight European and North American institutions, corroborated D30% and pre-RT dental extraction as key predictors while adding V70Gy and smoking status to the final model, achieving AUCs of 0.67–0.69 across training, test, and external validation cohorts (15). Model calibration was notably strong in the large population-based external validation cohort (Brier score 0.077, Log Loss 0.281), reinforcing the prognostic signal carried by these variables at a population level (15). It is important to note that calibration has been improving, and moderate AUCs can still be useful clinically in risk-stratification contexts.

Moving beyond binary endpoint modelling, the Weibull Accelerated Failure Time (WAFT) model developed by the MD Anderson Head and Neck Symptom Working Group (16) on 1,129 patients introduced a time-to-event framework that characterised the conditional probability of ORN across the full survivorship trajectory. The model identified D25% as a strongly significant predictor (Adjusted Time Ratio 0.88 per Gy increment, p<0.005), with each additional Gray associated with a 12% shortening of the time to ORN onset. Pre-RT dental extraction and male sex each accelerated ORN development, though without reaching conventional statistical thresholds, likely reflecting residual power constraints rather than absence of effect (16). The WAFT model achieved internal Harrell’s C-index of 0.72 at 72 months and was externally validated on an independent cohort from Guy’s and St Thomas’ NHS Foundation Trust. A complementary online graphical user interface now allows clinical users to input patient-specific parameters and visualise longitudinal risk trajectories, representing a meaningful step towards clinical integration (16).

Deep learning approaches have also been explored, most notably via a 3D DenseNet-40 convolutional neural network (3D-mDN40) trained on mandibular radiation dose distribution maps and clinical variables, which was externally validated by Humbert-Vidan et al. [16]. Encouragingly, this model demonstrated AUCs of approximately 0.70 at internal validation and 0.66 at external validation, comparable to logistic regression-based DVH models. This suggests that, while spatial dose information holds promise and have seen significant advances with further research, the incremental discriminative gain over DVH-based approaches has not yet been demonstrated sufficiently for broader adoption (17).

### Persistent methodological limitations

Despite these achievements, several interconnected methodological limitations undermine the clinical utility of existing NTCP models for ORN.

#### Cohort homogeneity and selection bias

The majority of ORN NTCP models have been derived from single-institution retrospective cohorts, most prominently at MDACC, where patient demographics, RT protocols, dental assessment pathways, and clinical follow-up intensity reflect institutional practice rather than population-level variation (14, 16). Even the PREDMORN consortium, despite its geographical breadth across eight institutions, relied predominantly on a 2:1 matched case-control design anchored to available ORN cases, introducing selection bias and artificially inflating ORN prevalence relative to the true population incidence (16). The consequence is that models trained on matched cohorts may perform poorly when applied to unselected clinical populations, a limitation clearly observed in PREDMORN when moving from matched to population-based validation cohorts (15). Large-scale prospective population-based data collection, spanning diverse patient groups, healthcare systems, and socioeconomic contexts, remains conspicuously absent from the ORN modelling literature.

#### DVH reduction and loss of spatial information

All established ORN NTCP models rely fundamentally on dose-volume histogram (DVH) metrics. The DVH effectively assumes a spatially invariant dose-response relationship within the mandible, an assumption that is almost certainly incorrect given the anatomical heterogeneity of mandibular bone, its regional vascular supply, and the proximity of dentition and alveolar bone to radiation fields (17). Van Dijk et al. acknowledged this limitation in the context of oral cavity cancer, where more homogeneous mandibular dose distributions reduced the discriminative power of DVH parameters (14). Deep learning approaches trained on three-dimensional dose distribution maps represent a methodological advance, but have yet to demonstrate significant performance benefits over DVH models in externally validated studies, and face practical challenges around data standardisation, segmentation variability, and computational accessibility across clinical sites (17).

#### Inadequate temporal modelling

Conventional NTCP models treat ORN as a binary endpoint observed at a single fixed time point, ignoring the right-censored, time-varying nature of ORN risk across the survivorship continuum. This is epidemiologically unjustifiable: ORN can develop at any point from weeks to years post-RT, with a median time to diagnosis of approximately 17–20 months in large cohort (14, 16). Furthermore, patients who survive longer are at progressively accumulating risk. The WAFT model represents a significant conceptual advance (16), but its external validation cohort was limited to a maximum follow-up of under 100 months compared to the training cohort’s timespan exceeding 200 months, limiting conclusions about long-term risk characterisation. Competing risk modelling remains largely unexplored and consequently represents a limitation to external validity.

#### Inconsistent and limited model performance

Reported AUC values across the ORN NTCP literature cluster in the 0.65–0.79 range, reflecting moderate discriminative ability that falls below the threshold typically considered necessary for clinical decision support in individual patients. PREDMORN’s test AUC of 0.69 (15) and the external validation AUCs of approximately 0.64–0.66 for deep learning models (17) are honest reflections of the current state of the art. While calibration has improved with larger and more diverse cohorts, discrimination remains constrained by the limited explanatory power of the variables currently included in models.

## A strategic framework for clinical translation

We propose three interconnected strategic priorities that, taken together, would substantially advance the clinical utility of NTCP models for ORN. These are not independent research silos but complementary components of an integrated evidence-generation programme.

### Expansion to wider, prospectively recruited, geographically diverse cohorts

The most fundamental constraint on current NTCP models is the quality and representativeness of the underlying data. A strategic priority must therefore be the systematic construction of prospective, population-based, multi-institutional cohorts that reflect the full diversity of HNC populations and treatment settings in which ORN NTCP models will ultimately be applied.

Existing consortium infrastructure (most notably PREDMORN, which currently encompasses eight institutions across North America and Europe) provides a platform for this expansion (15). However, three specific limitations require resolution. First, existing consortia are almost entirely confined to high-income countries with advanced RT infrastructure. Data from populations treated in lower- and middle-income settings, where RT techniques, fractionation schedules, and supportive care provision differ substantially, are entirely absent. This limits both the generalisability of derived models and the equity of any clinical tools built upon them. Second, consortium data collection in PREDMORN and related initiatives has been retrospective. This results impacts on data completeness, variable diagnostic parameters, and variable outcome ascertainment across sites (15). A prospective registry (while difficult to achieve), would support the implementation of a standardised minimum dataset. Third, the control-case matching paradigm (necessary to address ORN’s low prevalence in retrospective settings) should be superseded by population-based consecutive cohort recruitment wherever feasible, as case-matched designs systematically distort the representation of tumour subsites, dose distributions, and clinical risk factor distributions relative to real-world populations (15).

The publicly available longitudinal dataset published by West et al. (18), comprising 1,129 HNC patients with detailed demographic, clinical, and dosimetric data alongside structured follow-up and time-to-ORN events, exemplifies the data infrastructure that should become a minimum standard for participating institutions. The accompanying WAFT-based online graphical user interface (16) demonstrates that such datasets can support not only model development but also accessible clinical decision support tools, provided they are founded on sufficiently large and representative training populations.

Cohort expansion also enables investigation of interactions between patient-level factors and ORN risk that are currently underpowered in existing datasets. The role of HPV status is a paradigmatic example: despite the clinical importance of HPV-associated OPC as the fastest-growing HNC subtype, 81–89% of patients in the van Dijk and PREDMORN cohorts had unknown HPV/p16 status (14, 16, 18), effectively precluding analysis of what may be a clinically significant moderating variable. Similarly, race and ethnicity, socioeconomic status, body composition, and comorbidity burden are largely absent from existing models despite plausible biological and clinical relevance.

### Systematic characterisation of the influence of differing radiotherapy protocols

The ORN NTCP literature has developed in a context dominated by intensity-modulated radiotherapy (IMRT) and, to a lesser extent, volumetric-modulated arc therapy (VMAT), at institutions with relatively standardised prescription protocols. This narrow range of practice severely limits the generalisability of dose-response relationships to the full breadth of RT techniques and fractionation schedules encountered in contemporary HNC practice.

Several dimensions of RT protocol variation demand systematic characterisation. Fractionation is the most obvious scenario where this is applicable. Standard IMRT regimens deliver 2.0–2.12 Gy per fraction across 33 fractions, but hypofractionated schedules and accelerated regimens are increasingly employed in HNC treatment, with biological dose equivalencies at the mandible that may differ substantially from conventionally fractionated plans even when physical dose metrics appear similar (15). Furthermore, the radiotherapy delivery plan can vary significantly between centres introducing yet more heterogeneity. Additionally, PREDMORN explicitly noted that its inability to apply voxelwise EQD2 fractionation correction, due to the availability of only precalculated DVH summaries, precluded rigorous inter-institutional dose comparison, and that prescribed fraction size was instead included as a categorical covariate (15). It is clear, therefore, that dose distributions and site specific doses can vary significantly, impeding the clinical utility and generalisability of NTCP models.

Proton therapy represents a fundamentally distinct dosimetric environment that has received limited investigation in ORN NTCP modelling. Intensity-modulated proton therapy (IMPT) offers the potential for superior mandibular sparing in appropriately selected patients, but the ORN risk-dose relationship for proton beams may differ from that established for photon IMRT, and existing NTCP models cannot be assumed to transfer across modalities without explicit validation. The MDACC dataset included only 17 patients treated with IMPT in the control group and four in the ORN group (18). This is a sample size wholly insufficient to characterise a modality-specific dose-response relationship.

Simultaneous integrated boost (SIB) versus sequential boost regimens, the use of concomitant radiosensitisers such as nimorazole (as employed in Danish DAHANCA protocols) (17), and institutional variation in target volume delineation and normal tissue planning constraints collectively introduce dose heterogeneity that is not captured by DVH summary statistics. Future multi-institutional NTCP studies should explicitly stratify analyses by RT modality and fractionation regimen, and where possible use biologically equivalent dose (EQD2) metrics applied at the voxel level, facilitating meaningful cross-protocol comparison.

The interface between RT technique and ORN risk also intersects with evolving treatment de-escalation paradigms in HPV-associated OPC. If reduced-dose or reduced-volume RT regimens gain wider adoption in this population (as several ongoing clinical trials are exploring) it will be essential to understand whether the mandibular dose-response relationships calibrated on conventional-dose cohorts remain valid at lower dose levels, or whether threshold effects alter the shape of the dose-toxicity curve at doses below those currently represented in training data.

### Embedding granular, longitudinal oral health and dental treatment data

Of the three strategic pillars proposed here, the integration of comprehensive oro-dental data into NTCP modelling arguably represents the greatest unrealised opportunity, and the most significant current gap between the biological plausibility of ORN pathogenesis and the variables included in predictive models.

Pre-RT dental extraction has consistently emerged as a significant independent predictor of ORN across all major modelling efforts (14–16). Both pre-RT and post-RT dental extractions are recognised to influence the risk for ORN, including factors such as number of dental extractions, site of dental extractions, pre-existing infection, pre-existing oral health, the need for bone removal during extraction, and smoking status during dental extraction (19–22). Van Dijk et al. observed an odds ratio of 1.67 for dental extraction in their primary NTCP model (14), and PREDMORN confirmed this association across eight institutions with a statistically significant univariable p-value of 0.026 (15). The WAFT model identified dental extraction as accelerating ORN onset by approximately 27% (16). Yet in every case, dental extraction has been operationalised as a simple binary variable (extraction yes/no within a defined pre-RT window), discarding potentially critical granular information about extraction site, number and type of teeth extracted, the interval between extraction and RT commencement, the adequacy of socket healing, and whether extraction was performed for therapeutic versus prophylactic indications.

The PREDMORN consortium identified that the median time from extraction to RT start was significantly shorter in ORN cases than controls (20.0 vs 28.0 days), and that this difference was statistically significant (p=0.002) (15). Despite this, variable was not formally incorporated into the NTCP model due to the extent of missing data. This illustrates precisely the structural problem: when oro-dental data is collected retrospectively from clinical records in which it was not systematically recorded, the resulting missingness is non-random and the derived variables are inevitably crude. A dedicated prospective oro-dental assessment protocol, integrated into the pre-treatment RT pathway and recorded in a standardised digital format linkable to the RT planning dataset, would transform the quality of dental data available for modelling (23).

Beyond pre-RT extractions, several oro-dental parameters with strong biological plausibility as ORN risk modifiers are entirely absent from existing NTCP models. Periodontal bone loss*has been identified as an independent ORN risk factor at Memorial Sloan Kettering (14), but the small sample size precluded formal modelling. Post-RT dental extractions, which carry a substantially higher ORN risk than pre-RT extractions due to impaired osseous healing in irradiated bone, are not assessed in most studies because post-RT dental care is largely delivered outside the patient follow-up and often also outside the treating oncology institution. Radiation caries, which is a recognised and prevalent risk in irradiated patients carries a risk for dental extractions, represents an intermediate outcome that could serve as a time-updated covariate in longitudinal risk models; the DMFS160 scoring system developed specifically for post-radiation caries provides a quantitative instrument for this purpose (14). Oral hygiene, dentition status at baseline (edentulous versus dentate), and prosthetic rehabilitation status (particularly the use of implant-supported prostheses in irradiated bone) are all clinically recognised risk modifiers that remain outside the scope of current models.

There is also a compelling case for the integration of systemic indices of oro-dental health. Salivary function declines substantially following RT to the major salivary glands and drives accelerated caries progression, periodontal disease, and mucosal fragility. Salivary gland doses are available in only a minority of patients in existing ORN datasets (15), and formal sialometry data are almost entirely absent. Given that xerostomia is both a treatment toxicity and a mediating pathway for ORN, its inclusion as a time-updated covariate in longitudinal ORN models is both scientifically justified and practically feasible if prospective study methods are utilised.

Realising this pillar requires structural investment in two areas. First, multidisciplinary care pathways that formally embed dental oncology assessment (including standardised recording of periodontal status, caries activity, extraction history, and salivary function_ at defined pre- and post-treatment time points. This should be treated as a clinical quality standard rather than a research add-on, ensuring data completeness that retrospective approaches cannot achieve. Second, electronic health record systems should be configured to capture oro-dental data in structured, extractable formats that can be linked to RT dosimetric datasets without manual re-abstraction. The use of validated instruments such as the DMFS160 for caries monitoring (14) and formal sialometry protocols would enable longitudinal orodental data to function as dynamic covariates in time-to-event models, capturing the evolving biological environment within which ORN risk accumulates.

## Integrating the strategic pillars: towards a spatio-temporal, multi-modal NTCP framework

The three pillars described above are individually necessary but collectively insufficient if pursued in isolation. Their integration points towards a more ambitious methodological vision: a spatio-temporal, multi-modal NTCP framework that combines large, representative, prospectively collected cohorts; protocol-stratified dosimetric data referenced to biologically equivalent dose; and longitudinal oro-dental covariates within a time-to-event modelling structure.

The conceptual architecture for such a framework already exists within the ORN literature. The WAFT model (16) provides a temporal scaffolding that can accommodate time-updated covariates. Deep learning approaches applied to three-dimensional dose distribution maps (15) offer spatial specificity beyond DVH summaries, and while external validation results to date are modest, the approach is theoretically superior to DVH reduction for organs where spatial dose heterogeneity is clinically relevant. Machine learning methods (including random forests, gradient boosting, and neural networks_ have demonstrated utility in HNC toxicity prediction more broadly (24) and could leverage the higher-dimensional datasets that prospective multi-institutional collection would generate. The PREDMORN consortium’s planned second-phase analysis, focussing on imaging-based spatial dosimetric modelling (15), aligns directly with this vision.

Amalgamated learning approaches, already piloted in radiomic modelling for HNC outcomes (24), offer a practical solution to the data governance challenges that have historically constrained multi-institutional data aggregation, particularly across jurisdictions with differing data protection legislation. The PREDMORN model validation study employed a federated approach for its population-based external validation cohort (15), demonstrating feasibility. Extending federated infrastructure to cover the full model development cycle would enable participation of institutions that cannot share patient-level data externally, substantially expanding the effective cohort size available for training.

Standardisation remains a prerequisite. Inconsistent ORN grading systems (with Notani, Tsai, CTCAE, and the newer ClinRad system all represented across PREDMORN institutions (15, 25–28) have necessitated binary endpoint operationalisation and precluded severity-stratified modelling that would be clinically more informative. The adoption of the recently endorsed ClinRad classification by the ISOO-MASCC-ASCO guidelines (29) provides an opportunity to harmonise outcome ascertainment prospectively. Similarly, standardised mandible segmentation protocols [replacing the heterogeneous institutional approaches documented in PREDMORN (15)] and uniform DVH extraction methodologies would reduce the inter-institutional dosimetric variability that currently degrades model calibration.

## Implications for clinical practice and research governance

A clinically implemented ORN NTCP model would have substantive implications for treatment planning, patient counselling, and survivorship surveillance. In the treatment planning context, mandible dose constraints derived from validated NTCP models could be incorporated as formal planning objectives. This would be analogous to existing parotid gland dose constraints for xerostomia prevention. The online WAFT-based calculator (16) already enables risk-stratified surveillance recommendations: a patient with a D25% of 60 Gy, history of pre-RT extractions, and male sex could be assigned to enhanced post-RT oro-dental monitoring with earlier referral thresholds, while a patient with lower mandibular dose exposure and an intact dentition might appropriately be managed within standard surveillance pathways.

The decision-support utility of NTCP models also extends to treatment modality selection. NTCP-guided selection of patients for proton versus photon therapy requires validated ORN NTCP models for proton therapy to ensure that mandibular sparing advantages are not offset by unexpected toxicity at alternative dose distributions (14). This remains an area of significant unmet need.

From a research governance perspective, the strategic framework proposed here necessitates investment in multi-disciplinary collaboration infrastructure that bridges radiation oncology, dental oncology, medical physics, and data science. Existing consortia such as PREDMORN provide a template, but their sustainability and scalability depend on dedicated funding mechanisms, robust data governance frameworks, and the active participation of dental oncology as a co-equal partner in study design—not merely as a source of retrospectively extracted clinical variables. There are several obstacles to this, including: data protection constraints, inter-institutional differences in ORN grading, variability in mandible segmentation, incomplete HPV annotation, and the challenge of capturing post-RT dental outcomes when care occurs outside the treating centre. Overcoming these may not be possible in their entirety due to their structural nature; however, minimising their impact is vital to ensuring the feasibility of future research.

## Conclusion

NTCP modelling for ORN in HNC has reached a methodological inflection point. The foundational work of the past five years, from the first validated single-institution NTCP model through multi-institutional consortium development to time-to-event frameworks and deep learning approaches, has established that quantitative ORN risk prediction is scientifically feasible and clinically meaningful. The consistent identification of mandibular dose metrics and pre-RT dental extraction as independent predictors across diverse modelling approaches and populations provides a robust empirical foundation.

The challenge now is not one of concept but of execution. Translating these models into tools that reliably inform individual clinical decisions requires a step-change in the quality, scale, and representativeness of underlying data. A systematic strategy to account for the full range of RT techniques and fractionation regimens encountered in contemporary practice; and the formal integration of oro-dental health as a dynamic, longitudinally characterised model input rather than a retrospective binary covariate. The three strategic pillars proposed here (wider cohort expansion, RT protocol characterisation, and oro-dental data embedding) are individually driven by specific evidence gaps and collectively oriented towards a spatio-temporal, multi-modal predictive framework that reflects the biological complexity of ORN and the clinical heterogeneity of the populations at risk. The individual data fields collected for such research are important to consider, however it is outwith the present publication to propose an optimal array of variables. Rather, this should be considered by a working group incorporating key members from a range of specialities, in a similar manner to other publications on reporting standards.

The growing population of long-term HNC survivors, many of them young adults with HPV-associated disease, deserves prediction tools calibrated to their lifetime risk profile. Meeting that obligation is both a scientific challenge and an ethical imperative.
